# Identifying potential pharmacological targets and mechanisms of vitamin D for hepatocellular carcinoma and COVID-19

**DOI:** 10.3389/fimmu.2022.985781

**Published:** 2022-08-17

**Authors:** Yongbiao Huang, Ye Yuan, Sheng Chen, Duo Xu, Lingyan Xiao, Xi Wang, Wan Qin, Bo Liu

**Affiliations:** ^1^ Department of Oncology, Tongji Hospital, Tongji Medical College, Huazhong University of Science and Technology, Wuhan, China; ^2^ Department of Gastroenterology, Tongji Hospital, Tongji Medical College, Huazhong University of Science and Technology, Wuhan, China; ^3^ Department of general surgery, Shangrao People's Hospital, Shangrao, China

**Keywords:** COVID-19, hepatocellular carcinoma, vitamin D, network pharmacology, molecular docking, prognosis

## Abstract

Coronavirus disease 2019 (COVID‐19) is a severe pandemic that has posed an unprecedented challenge to public health worldwide. Hepatocellular carcinoma (HCC) is a common digestive system malignancy, with high aggressiveness and poor prognosis. HCC patients may be vulnerable to COVID-19. Since the anti-inflammatory, immunomodulatory and antiviral effects of vitamin D, we aimed to investigate the possible therapeutic effects and underlying action mechanisms of vitamin D in COVID-19 and HCC in this study. By using a range of bioinformatics and network pharmacology analyses, we identified many COVID-19/HCC target genes and analyzed their prognostic significance in HCC patients. Further, a risk score model with good predictive performance was developed to evaluate the prognosis of HCC patients with COVID-19 based on these target genes. Moreover, we identified seven possible pharmacological targets of vitamin D against COVID-19/HCC, including HMOX1, MB, TLR4, ALB, TTR, ACTA1 and RBP4. And we revealed the biological functions, signaling pathways and TF-miRNA coregulatory network of vitamin D in COVID-19/HCC. The enrichment analysis revealed that vitamin D could help in treating COVID-19/HCC effects through regulation of immune response, epithelial structure maintenance, regulation of chemokine and cytokine production involved in immune response and anti-inflammatory action. Finally, the molecular docking analyses were performed and showed that vitamin D possessed effective binding activity in COVID-19. Overall, we revealed the possible molecular mechanisms and pharmacological targets of vitamin D for treating COVID-19/HCC for the first time. But these findings need to be further validated in actual HCC patients with COVID-19 and need further investigation to confirm.

## Introduction

Coronavirus disease 2019 (COVID‐19), an ongoing global pandemic caused by severe acute respiratory syndrome coronavirus 2 (SARS-CoV-2), has posed a substantial challenge to healthcare systems around the world (1, 2). As of March 4, 2022, with 440,807,756 confirmed COVID-19 cases and 5,978,096 deaths reported globally (3). Although multiple COVID-19 vaccines have been developed and mass vaccinations have been undertaken, the number of infections is still continuously increasing (4). Besides, some antiviral drugs have been applied for treating COVID-19, such as remdesivir, but due to its high price and the need for intravenous administration, it has not been widely used (5). Thus, it is essential to screen effective, inexpensive and readily available drugs against COVID-19. Additionally, cancer patients, such as hepatocellular carcinoma (HCC), were reported to be at higher risk of COVID-19 infection and developing severe complications than those noncancer people (6–8). HCC is a common digestive system malignancy, with high aggressiveness and poor prognosis. Globally, HCC has the sixth highest incidence among all cancers, and the incidence has been continuously increasing in recent years (9, 10). HCC patients infected with COVID-19 will be very difficult to treat, because there is a lack of effective drugs that can improve immunity and against both COVID-19 and HCC. Therefore, screening effective therapeutic agents for such patients is important.

Vitamin D is a fat-soluble vitamin, including two major forms: vitamin D2 and vitamin D3. The predominant source of vitamin D in humans is derived from skin synthesis (VD3) and dietary intake (VD2 or VD3). To exert biological activity, vitamin D is converted to 25-hydroxyvitamin D (25(OH)D, circulating form) through hydroxylation in the liver, and then hydroxylated to the 1,25-dihydroxyvitamin D (1,25(OH)2D, active form) in the kidneys (11). The classical physiological function of vitamin D is to maintain calcium and phosphorus homeostasis and regulate bone metabolism (12). Recently, vitamin D has also been found to have multiple nonclassical functions including immunomodulation, anti-inflammation, anti-virus and anti-tumor (13–15). Numerous reports have indicated that vitamin D deficiency is associated with more severe COVID-19, and patients with low levels of vitamin D have higher mortality (16, 17). Vitamin D could inhibit NF-κB signaling, reduce the production of various pro-inflammatory cytokines, and thereby might help suppress cytokine storm in COVID-19 (18, 19). Furthermore, vitamin D exhibits anti-hepatocarcinogenic effects by inhibiting tumor cell proliferation, invasion and promoting apoptosis (20). However, the pharmacological targets and molecular mechanisms of vitamin D against COVID-19 in HCC patients are remain be fully studied.

In this study, we used the network pharmacology and bioinformatics approaches to investigate the prognostic value of COVID-19 related genes in HCC patients, and further explore the possible anti-COVID-19/HCC mechanisms of vitamin D. Our findings provide some new insights into vitamin D in the treatment of COVID-19/HCC.

**Figure 1:**
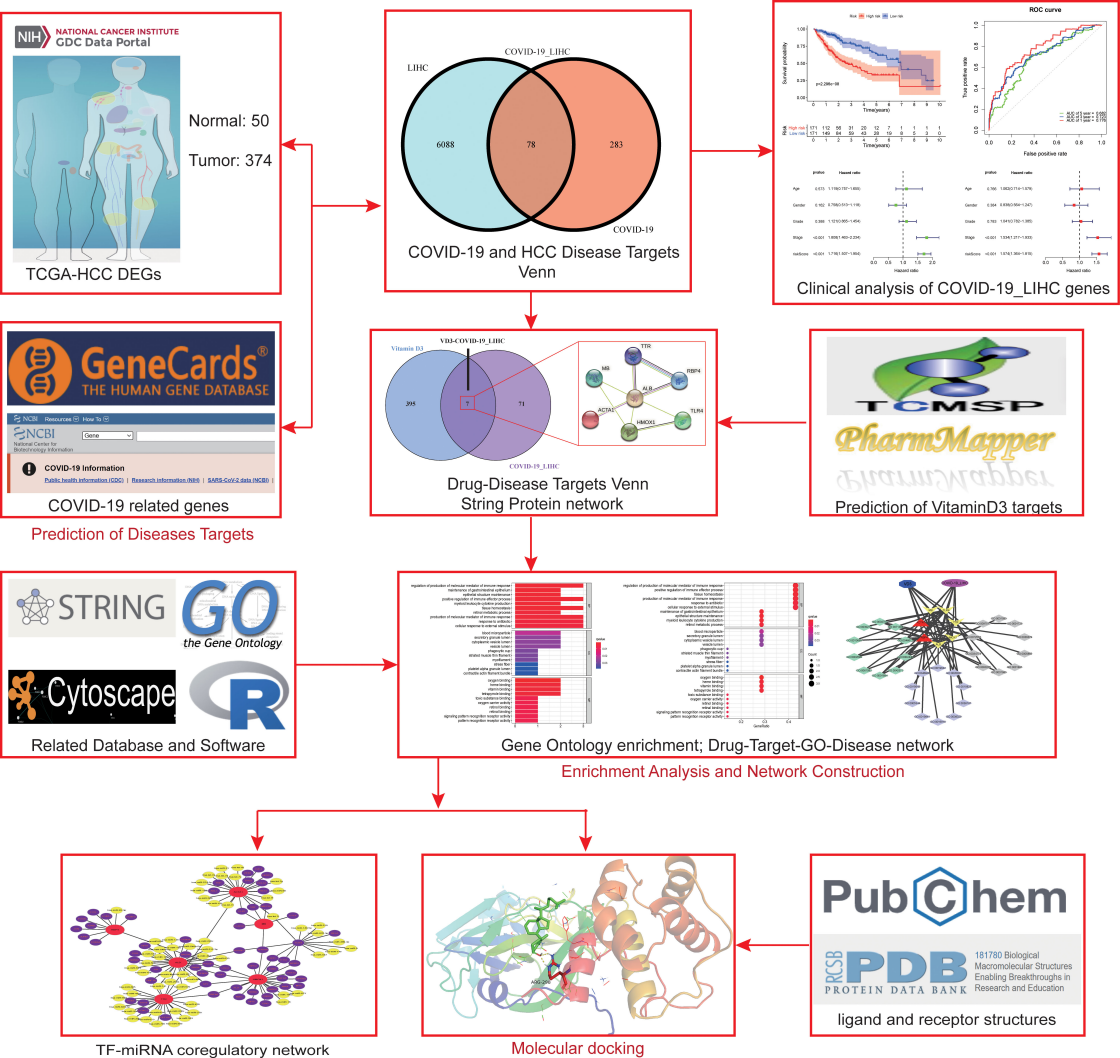
Workflow of the whole study.

The entire workflow of this study was summarized in a visible graphical abstract. (**Figure 1**).

## Materials and methods

### Data collection and identifying COVID-19/HCC related genes

This investigation was a retrospective cohort study, which was approved by The Medical Ethics Committee of Tongji Hospital, Tongji Medical College, Huazhong University of Science and Technology (TJ-IRB20200409). The transcriptome profiles and clinical data of HCC patients were downloaded from The Cancer Genome Atlas (TCGA) database, then the differentially expressed genes were identified using the ‘edgeR’ R package with |log2 fold change (FC)| >1.0 and false discovery rate (FDR) <0.05 (21). Besides, the COVID-19 related genes were obtained from the Genecard database and NCBI gene module (22). Finally, we took the intersection of these genes and obtained the overlapping targets in HCC and COVID-19, and these overlapping genes were displayed in a volcano plot.

### Prognostic analysis of HCC and COVID-19 related genes

The association of COVID-19/HCC related genes with survival in HCC patients was analyzed using univariate Cox regression by the ‘survival’ R package, and the protein-protein interaction (PPI) network of the identified prognostic genes were explored in the STRING database (version 11.0). Moreover, a risk score model was further constructed based on multivariate Cox proportional hazards regression model, and the predictive ability of this model was evaluated through receiver operating characteristic (ROC) curves (10). Patients were divided into high-risk (n=171) and low-risk (n=171) groups based on the median risk score, and their overall survival rates were compared through the Kaplan–Meier method with the log-rank test. Additionally, the independent prognostic value of this risk model was analyzed using univariate analysis and multivariate regression analyses. Finally, we validated the performance of the prognostic model in an external validation set (ICGC, LIRI-JP project).

### Acquiring the pharmacological targets of vitamin D against COVID-19/HCC

We collected and screened all pharmacological targets of vitamin D3 from freely accessible online databases designed to identify potential pharmacological targets for the given small molecules, including the Traditional Chinese Medicine Database and Analysis Platform (TCMSP) and PharmMapper Server (23, 24). The overlapping target genes of vitamin D3 in COVID-19/HCC were further acquired, and the PPI network was generated in the STRING database.

### Enrichment analysis and interaction network visualization

The gene ontology (GO) and KEGG enrichment analyses of the overlapping target genes of vitamin D in COVID-19/HCC was performed using the ‘ClusterProfiler’ R language package, and the enrichment results were visualized using the ‘GOplot’ R package, the p-value and q-value were set at 0.05 (25). The drug-target-GO function-disease interaction network was constructed using Cytoscape software (version 3.7.1) to illustrate the biological function of target genes of vitamin D treatment in COVID-19/HCC (26).

### TF-miRNA coregulatory network

TF-miRNA coregulatory interaction information was obtained from the RegNetwork repository, and the TF-miRNA coregulatory network was generated by using NetworkAnalyst (https://dev.networkanalyst.ca), and further visualized through Cytoscape software. NetworkAnalyst is a comprehensive platform for interaction network analysis, including protein-protein interactions, gene regulatory networks and gene coexpression networks (27).

### Molecular docking

The 2-dimensional molecular structure of vitamin D3 was obtained from the PubChem database (https://pubchem.ncbi.nlm.nih.gov/) (28), and its 3-dimensional structure was generated and optimized by the MM2 force field in ChemBioOffice software (version 2014) (29). Finally, the output ligand file of vitamin D3 was saved as mol2 format. The protein structures of COVID-19 associated proteins were obtained from the PDB database (http://www.rcsb.org/pdb) (30). Then, all water molecules and ligands were removed from the structures using PyMOL software and saved as PDB files. The original protein receptor and ligand files were converted to PDBQT file format with AutoDockTools 1.5.6, which could be recognized by the Autodock Vina program for subsequent docking experiments (31). Finally, all docking results were displayed and analyzed by PyMOL software (32).

### Statistical analysis

Statistical analyses in this work were conducted by R software (version 3.6.3). The survival between different groups were compared through the Kaplan-Meier method with the log-rank test. Univariate and multivariate Cox regression were applied to compare the impact of the risk score model and other clinical characteristics on survival. The risk scores of subgroups classified by different clinical characteristics were compared with Wilcoxon test. The P < 0.05 was regard to have statistical significance.

## Results

### Identification of COVID-19/HCC targets

**Figure 2:**
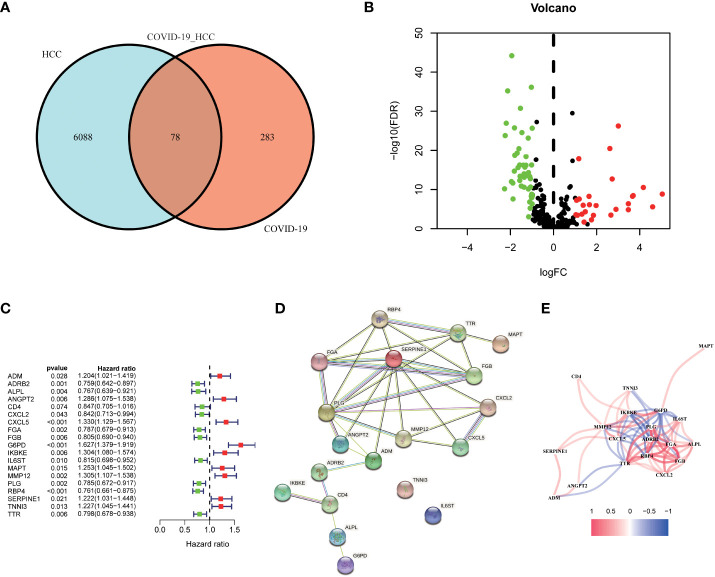
Identification of the candidate genes in COVID-19/HCC. **(A)** Venn diagram to identify the intersecting genes in COVID-19/HCC. **(B)** Volcano plot of the intersecting genes in HCC. **(C)** The results of the univariate Cox analysis presented in a forest plot. **(D)** The interaction network among the 19 COVID-19/HCC related genes. **(E)** The correlation network of the 19 COVID-19/HCC related genes.

First, we identified 6166 HCC-associated differentially expressed genes (DEGs) in TCGA database. Meanwhile, 361 COVID-19 associated genes were collected from the Genecard and NCBI databases through network pharmacology. The intersection of these two gene clusters was shown in **Figure 2A**, and 78 common genes in HCC and COVID-19 were identified. Further, the differential expression of these common genes was checked, of which 27 genes were found to be upregulated and 61 genes were downregulated in HCC (**Figure 2B**).

### Prognostic value of COVID-19/HCC associated genes

To explore the correlation between COVID-19/HCC associated genes and the prognosis of COVID-19/HCC patients, univariate and multivariate Cox analyses were performed on the 78 DEGs. First, 19 genes significantly associated with COVID-19/HCC were identified through univariate Cox analysis, including ADM, ADRB2, ALPL, ANGPT2, CD4, CXCL2, CXCL5, FGA, FGB, G6PD, IKBKE, IL6ST, MAPT, MMP12, PLG, RBP4, SERPINE1, TNNI3 and TTR (P <0.05, **Figure 2C** and **Table 1**). The interaction network of these genes was presented in **Figure 2D**, and the correlations between them were showcased in **Figure 2E**. Thereafter, a six-gene signature containing ALPL, ANGPT2, CD4, G6PD, SERPINE1, and TNNI3 was developed through multivariate Cox regression analysis (**Table 2**). The patient’s risk score was calculated based on the regression coefficient and expression of these six genes: Risk score = 0.250 * ANGPT2 Exp + 0.436 * G6PD Exp + 0.211* SERPINE1 Exp + 0.201 * TNNI3 Exp − 0.151 * ALPL Exp − 0.241 * CD4 Exp.

**Table 1:** Univariate Cox regression analysis of COVID-19/HCC related genes.

Symbol	HR	Lower 95% CI	Upper 95% CI	P-value
ADM	1.203567	1.020617	1.419311	0.027627
ADRB2	0.759201	0.642236	0.897468	0.00125
ALPL	0.767183	0.639181	0.920818	0.004431
ANGPT2	1.28599	1.07512	1.538218	0.005912
CD4	0.846686	0.705319	1.016386	0.074167
CXCL2	0.842264	0.713441	0.994348	0.042673
CXCL5	1.330195	1.129395	1.566696	0.000632
FGA	0.787212	0.679012	0.912653	0.001516
FGB	0.805357	0.689874	0.940173	0.006122
G6PD	1.626622	1.378565	1.919313	8.27e-09
IKBKE	1.303744	1.080161	1.573608	0.005722
IL6ST	0.815436	0.698339	0.952169	0.00989
MAPT	1.252519	1.044707	1.501668	0.014997
MMP12	1.304917	1.106879	1.538388	0.001529
PLG	0.784992	0.671732	0.917348	0.002326
RBP4	0.760531	0.660916	0.875159	0.000133
SERPINE1	1.221538	1.030646	1.447786	0.020992
TNNI3	1.227173	1.044765	1.441428	0.012655
TTR	0.797681	0.678232	0.938168	0.006312

HR, hazard ratio; CI, confidence interval.

**Table 2:** Multivariate Cox proportional hazards regression model.

Symbol	Coefficient	HR	Lower 95% CI	Upper 95% CI	P-value
ALPL	-0.15074	0.860072	0.718649	1.029326	0.100051
ANGPT2	0.250202	1.284285	1.058391	1.558391	0.011247
CD4	-0.2413	0.785603	0.647798	0.952723	0.014202
G6PD	0.436329	1.547018	1.298831	1.842629	1.01E-06
SERPINE1	0.21077	1.234628	1.038959	1.467147	0.016662
TNNI3	0.200939	1.22255	1.022867	1.461214	0.027212

HR, hazard ratio; CI, confidence interval.

**Figure 3:**
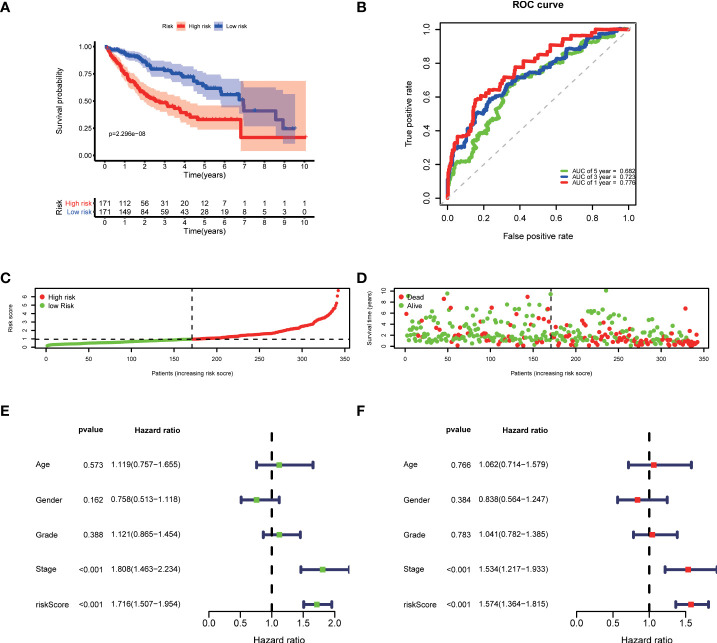
Development of the prognostic model based on COVID-19/HCC associated genes in the TCGA cohort. **(A)** Kaplan-Meier survival analysis between the high- and low-risk patients. **(B)** Time-dependent ROC curves at 1,3,5-years. **(C)** Distribution of risk scores, **(D)** survival status of each patient. **(E)** Univariate and **(F)** multivariate Cox regression analyses.

**Figure 4:**
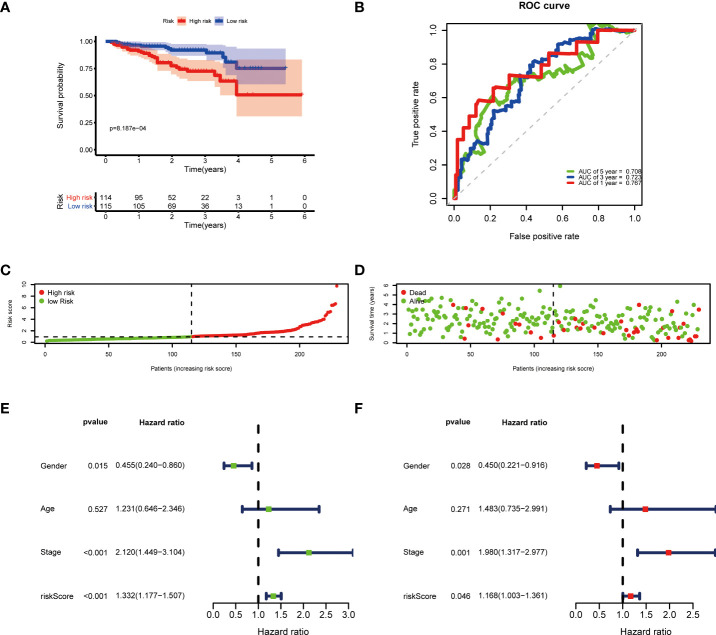
Validation of the prognostic model in the ICGC cohort. **(A)** Kaplan-Meier survival analysis between the high- and low-risk patients. **(B)** Time-dependent ROC curves at 1,3,5-years. **(C)** Distribution of risk scores, **(D)** survival status of each patient. **(E)** Univariate and **(F)** multivariate Cox regression analyses.

Then, according to the cut-off of the median risk score, the patients in TCGA cohort were stratified into high- and low-risk groups. In the Kaplan-Meier survival analysis, we found that patients in the high-risk group exhibited a significantly shorter overall survival compared with those in the low-risk group (**Figure 3A**). Time-dependent ROC curves of the six-gene signature were shown in **Figure 3B**, and the AUC values were 0.776 at 1 year, 0.723 at 3 years and 0.682 at 5 years. These results showed that the risk score could act as an effective prognostic indicator. Moreover, patients in high-risk group presented a higher probability of early death than those in the low-risk group (**Figure 3C, D**). Univariate and multivariate Cox analyses were applied among the available clinical characteristics to determine the independent prognostic value of the risk score for overall survival. We observed that the risk score was significantly correlated with the survival of HCC patients in univariate Cox regression analysis (HR= 1.716, 95% CI = 1.507-1.954, P< 0.001). Interestingly, after adjusting for other confounders, the risk score was still demonstrated as an independent predictor for survival in multivariate regression analysis (HR= 1.574, 95% CI = 1.364-1.815, P< 0.001). Moreover, the risk scores of patients were calculated using the same formula in the ICGC cohort, and we obtained similar results with the TCGA cohort (**Figure 4**). These results demonstrated a robust predictive performance of the six-gene prognostic signature.

### Clinicopathological analysis of the risk score model

**Figure 5:**
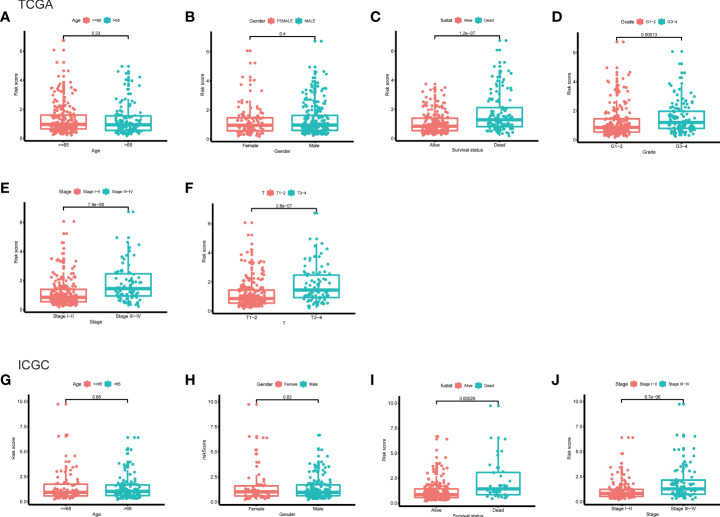
The differences of risk score between subgroups stratified by different clinical characteristic in the TCGA and ICGC cohorts. **(A)** Age, **(B)** gender, **(C)** survival status, **(D)** grade, **(E)** stage, **(F)** T classification in the TCGA cohort. **(G)** Age, **(H)** gender, **(I)** survival status, **(J)** stage in the ICGC cohort.

**Figure 6:**
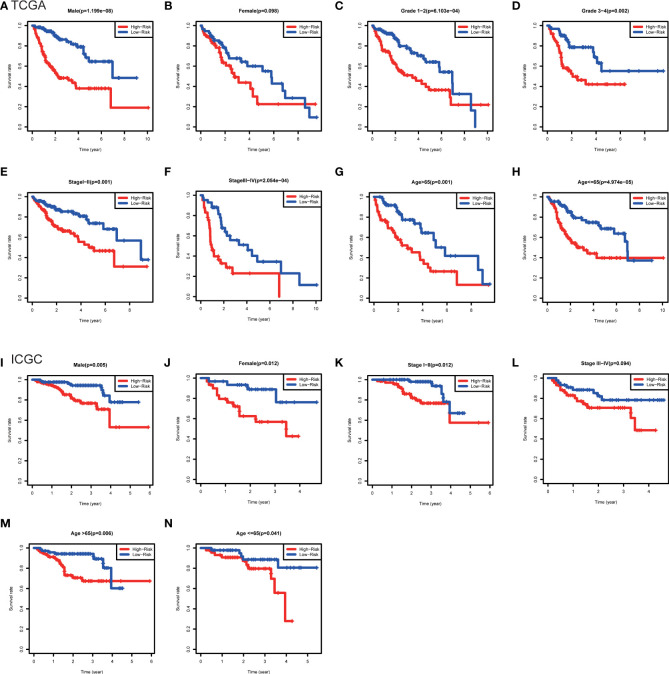
Kaplan-Meier survival analysis between subgroups stratified by different clinical characteristics in the TCGA and ICGC cohorts. **(A)** Male, **(B)** female, **(C)** grade 1-2, **(D)** grade 3-4, **(E)** stage I-II, **(F)** stage III-IV, **(G)** age>65, **(H)** age<=65 in the TCGA cohort. **(I)** male, **(J)** female, **(K)** stage I-II, **(L)** stage III-IV, **(M)** age>65, **(N)** age<=65 in the ICGC cohort.

Furthermore, the clinicopathological analysis of the risk score indicated that the risk score was not related to age and gender in both TAGA and ICGC cohorts (**Figures 5A, B, G, H**). Whereas, patients with poor clinical outcomes usually had higher risk scores (**Figure 5C, I**). Additionally, the risk score was closely associated with higher pathological grade, more advanced stage and larger tumor size of HCC (**Figures 5D–F, J**). The further Kaplan-Meier survival analysis stratified by different clinicopathologic features showed that the high-risk patients had a poor prognosis in all subgroups (**Figure 6**). The results demonstrated that the risk score model could predict survival for HCC patients without considering clinicopathologic features.

### Identifying intersection targets of vitamin D against COVID-19 and HCC

**Figure 7:**
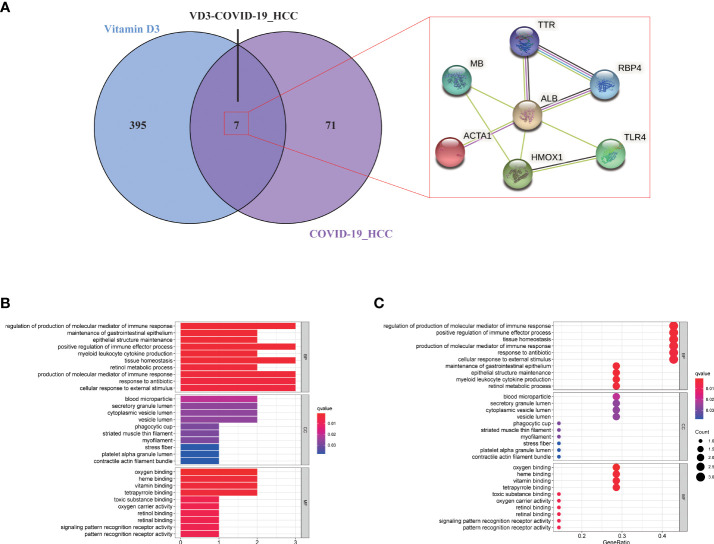
Functional enrichment analysis of vitamin D against COVID-19/HCC intersecting genes. **(A)** Venn diagram of vitamin D associated targets and COVID-19/HCC genes. **(B)** Bar plot and **(C)** bubble plot showing the results of GO enrichment analysis of vitamin D against COVID-19/HCC intersecting genes.

The pharmacological targets of vitamin D were determined by using TCMSP and PharmMapper databases, and 402 vitamin D-associated targets were identified. By taking the intersection of COVID-19/HCC genes and vitamin D-associated targets, we obtained seven overlapping genes (HMOX1, MB, TLR4, ALB, TTR, ACTA1 and RBP4) of vitamin D against COVID-19/HCC. Additionally, the PPI network of these intersection genes was constructed in the STRING database (**Figure 7A**). To explore the biological functions and pathways in which the seven intersection genes were involved, GO and KEGG enrichment analyses were conducted on these genes. The results indicated that vitamin D affects a series of biological processes, including regulation of production of molecular mediator of immune response, maintenance of gastrointestinal epithelium, epithelial structure maintenance, positive regulation of immune effector process, myeloid leukocyte cytokine production, tissue homeostasis, retinol metabolic process, response to antibiotic, cellular response to external stimulus, positive regulation of chemokine production, positive regulation of cytokine biosynthetic process, regulation of cytokine production involved in immune response (**Figures 7B, C** and **Table S1**). Whereas in the KEGG pathway analysis, only thyroid hormone synthesis and HIF-1 signaling pathway were significantly enriched (P < 0.05, **Figure S1**).

### TF-miRNA coregulatory network

**Figure 8:**
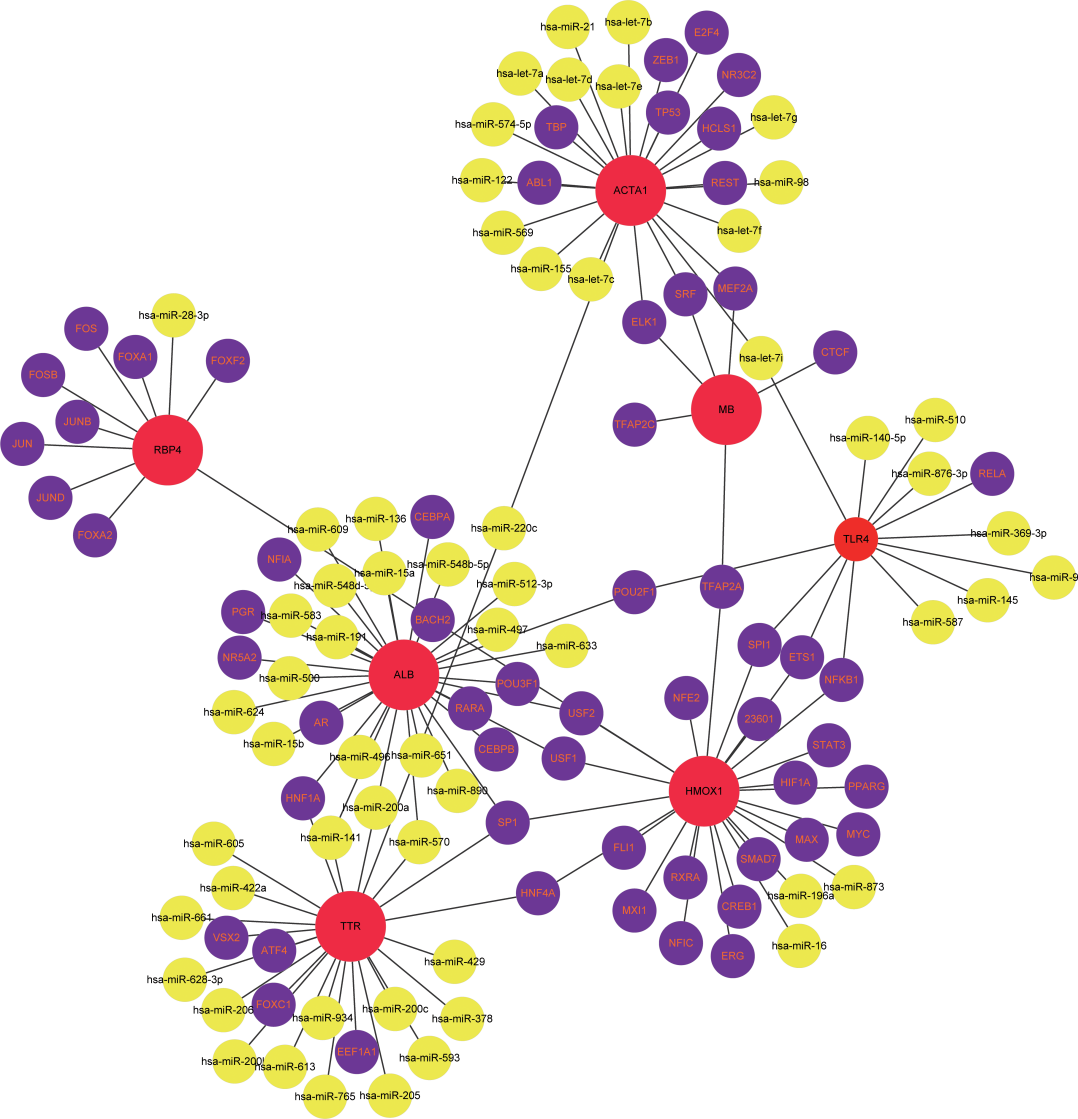
The TF-miRNA coregulatory network of vitamin D against COVID-19/HCC intersecting genes.

The TF-miRNA coregulatory network of the seven overlapping gene targets of vitamin D against COVID-19/HCC was analyzed using NetworkAnalyst. The TF-miRNA coregulatory network analysis provided information about TFs and miRNAs interaction with these target genes. These interactions might be responsible for regulating the expression of these target genes. The TF-miRNA-gene interaction network contains a total of 125 nodes and 138 edges, 59 TFs and 59 miRNAs that interacted with these seven overlapping target genes. In the network, ALB is regulated by 13 TFs and 19 miRNAs, HMOX1 is regulated by 23 TFs and 3 miRNAs, ACTA1 is regulated by 11 TFs and 15 miRNAs, TTR is regulated by 7 TFs and 18 miRNAs, TLR4 is regulated by 5 TFs and 8 miRNAs, RBP4 is regulated by 9 TFs and 1 miRNA, and MB is regulated by 6 TFs (**Figure 8**).

### Binding of vitamin D to COVID-19 and potential intersection targets

**Figure 9:**
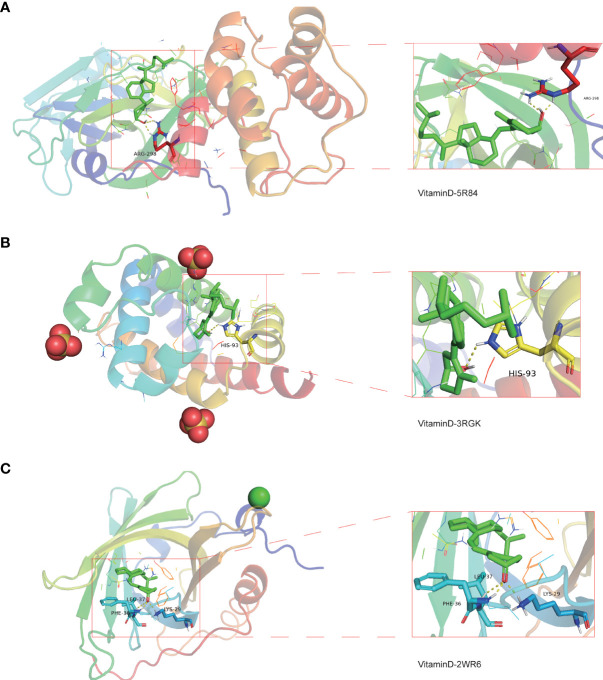
Molecular docking analysis for the binding of vitamin D to COVID-19 and the targets MB and RBP4. **(A)** The binding of vitamin D with SARS-CoV-2 main protease (PDB ID 5R84). **(B)** The binding of vitamin D with the 3RGK protein of MB. **(C)** The binding of vitamin D with the 2WR6 protein of RBP4.

To determine the possible binding of vitamin D with COVID-19 and the previously identified intersection targets, molecular docking analysis was carried out. We obtained the crystal structure of the COVID-19 main protease (PDB ID 5R84) from the PDB database, for subsequent molecular docking with vitamin D. The docking results demonstrated that vitamin D possessed good binding activity with the COVID-19 main protease and formed two hydrogen bonds with the amino acid residue ARG-298 (2.0 Å and 2.3 Å) of protein 5R84 (**Figure 9A**). Next, we further analyzed the possible binding of vitamin D with the seven COVID-19/HCC targets (HMOX1, MB, TLR4, ALB, TTR, ACTA1 and RBP4) identified previously and found that vitamin D only binds to MB and RBP4. The crystal structures of MB and RBP4 were also gathered from the PDB database with PDB IDs 3RGK and 2WR6 respectively. We found that hydrogen bonding between vitamin D and the protein MB acted on the amino acid residue HIS-93 (2.6 Å) (**Figure 9B**). Furthermore, vitamin D bound to the RBP4 protein by forming hydrogen bonds with the amino acid residues LEU-37 (2.6 Å), LYS-29 (2.0 Å) and PHE-36 (1.5 Å) (**Figure 9C**). These results showed the high-affinity between vitamin D with MB and RBP4.

## Discussion

COVID-19 is a serious, rapidly spreading infectious disease, that can be life-threatening (33). At this time of writing, with more than 400 million people infected with COVID-19 and more than 5.5 million died from COVID-19, and the numbers are still growing (3). The risk factors for COVID-19 infection and severe outcomes identified currently include male sex, older age, obesity, diabetes mellitus, cardiovascular disease and cancer (6, 8, 34). In particular, patients with cancer are more vulnerable to COVID-19 and contribute to adverse outcomes due to their low immunity and immunological dysfunction (7, 35). As per the latest global cancer statistics in 2020, the incidence of HCC ranks sixth, and the mortality ranks third among all cancers (9). Moreover, the COVID-19 pandemic is still raging, and HCC patients remain at an increased risk of COVID-19 infection. Thus, COVID-19 infection in HCC patients may lead to worse outcomes and amplify the risk of death.

In some previously published studies, vitamin D showed anti-proliferative and anti-invasive effects on HCC cells, which could inhibit HCC progression by inducing apoptosis, reducing oxidative stress and inflammation (36–39). Also, vitamin D is known to have direct antiviral and immunomodulatory properties (40, 41). Moreover, vitamin D deficiency is associated with an increased risk of both COVID‐19 and HCC (42). Thus, we hypothesize that vitamin D may exert potent pharmacological effects in HCC patients with COVID-19.

In the current study, we first collected 361 COVID-19 target genes and identified 6166 DEGs in HCC, and then further screened out 78 common genes of COVID-19 combined with HCC. Among these genes, 27 genes were upregulated and 61 genes were downregulated in HCC and/or COVID-19 patients. As per univariate and multivariate prognostic analyses, a few important genes, including ALPL, ANGPT2, CD4, G6PD, SERPINE1 and TNNI3, may serve as independent prognostic factors for HCC patients with COVID-19. Then, we developed a prognostic model based on the six genes to predict survival in patients with HCC and COVID-19, and the model exhibited good prediction capability in both independent cohorts without considering clinicopathologic features. Additionally, the prognostic model was significantly associated with HCC progression, which can used to screen and characterize different stages of HCC patients with COVID-19. This prognostic model predicts survival independently of gender, age and tumor stage, and the gender, stage and risk score were all influential factors for survival. Until now, the TNM staging system is still a very important tool for predicting the survival of HCC patients. Due to the excellent predictive ability of this prognostic model, it may be used as a supplement to TNM staging system.

Put together, these 78 intersection genes may be potential therapeutic targets of HCC and COVID-19. Further, we identified seven overlapping genes of vitamin D against HCC and COVID-19 using the network pharmacology approach, and the anti-COVID-19/HCC effects of vitamin D may be modulated by these molecules or genes, including HMOX1, MB, TLR4, ALB, TTR, ACTA1 and RBP4. There were significant differences in the expression of these genes. The HCC patients showed increased expression of MB and ACTA1, decreased expression of HMOX1, TLR4, ALB, TTR and RBP4. Moreover, the decreased TTR and RBP4 expression was relevant for worse prognosis of HCC patients. The MB gene encodes myoglobin, an oxygen−binding hemoprotein, which was reported to be ectopically expressed in different human cancer cell lines and cancer tissues (43). ACTA1 was identified as a biomarker for head and neck squamous cell carcinoma and colorectal cancer (44, 45). HMOX1 encodes heme oxygenase 1, a stress-inducible enzyme, that plays an essential role in oxidative stress response. It is known to have anti-inflammatory and immunomodulatory effects, and it may be a promising target for the treatment of COVID-19 (46). ALB is a tumor suppressor in HCC that can inhibit the proliferation of HCC cells and regulate the cell cycle (47). Besides, ALB knockdown promoted the migration and invasion of HCC cells through the upregulation of uPAR, MMP2, and MMP9 (48). TTR was regarded as an independent prognostic factor for HCC patients, and the low serum TTR levels were associated with poor prognosis (49). RBP4 was considered a novel biomarker for predicting HCC prognosis, and decreased expression of RBP4 indicated a worse prognosis and correlated with immune infiltration in HCC (50). MB, TLR4 and ALB are involved in coronavirus biology, and are associated with COVID-19 prognosis (51–53). The serum myoglobin was an effective predictor of the prognosis in COVID-19 hospitalized patients, the higher serum myoglobin levels were related to poor prognosis of COVID-19 patients (54). TLR4 was reported to be able to directly interact with SARS-CoV-2 spike protein and activates related inflammatory responses, whilst the TLR4-specific inhibitor resatorvid could completely block the secretion of IL1B induced by SARS-CoV-2 (55). These findings further suggested that these seven intersection genes might be potent pharmacological targets of vitamin D against HCC and COVID-19.

The GO and KEGG enrichment analyses based on these seven intersection genes showed that vitamin D exerts the anti-COVID-19/HCC effects directly *via* regulation of immune response, epithelial structure maintenance, regulation of chemokine and cytokine production involved in immune response, anti-viral and anti-inflammatory actions, as well as regulation of the HIF-1 pathway. Cytokine storm caused by SARS-CoV-2 infection is the main cause of death in COVID-19 patients, thus, the inhibition of cytokine production may be an important screening condition of effective COVID-19 drugs (56). HIF-1 pathway is involved in the regulation of oxidative stress and inflammation, and the activity of HIF-1 pathway may promote SARS-CoV-2 infection and affect a variety of physiological functions (57, 58). Therefore, inhibiting the activity or activation of HIF-1 pathway may be used to prevent COVID-19.

Lastly, we identified good binding activities of vitamin D with the 5R84 structure in COVID-19, the 3RGK structure in the target MB and the 2WR6 structure in the target RBP4 through molecular docking analysis, suggesting that vitamin D can effectively bind to specific proteins associated with SARS-CoV-2, and that vitamin D may be able to act on the MB and RBP4 to target COVID-19. Therefore, we believe that vitamin D supplementation may improve the efficacy of current antiviral therapy and immunotherapy for the treatment of COVID-19, or the combination of HCC with COVID-19.

**Figure 10:**
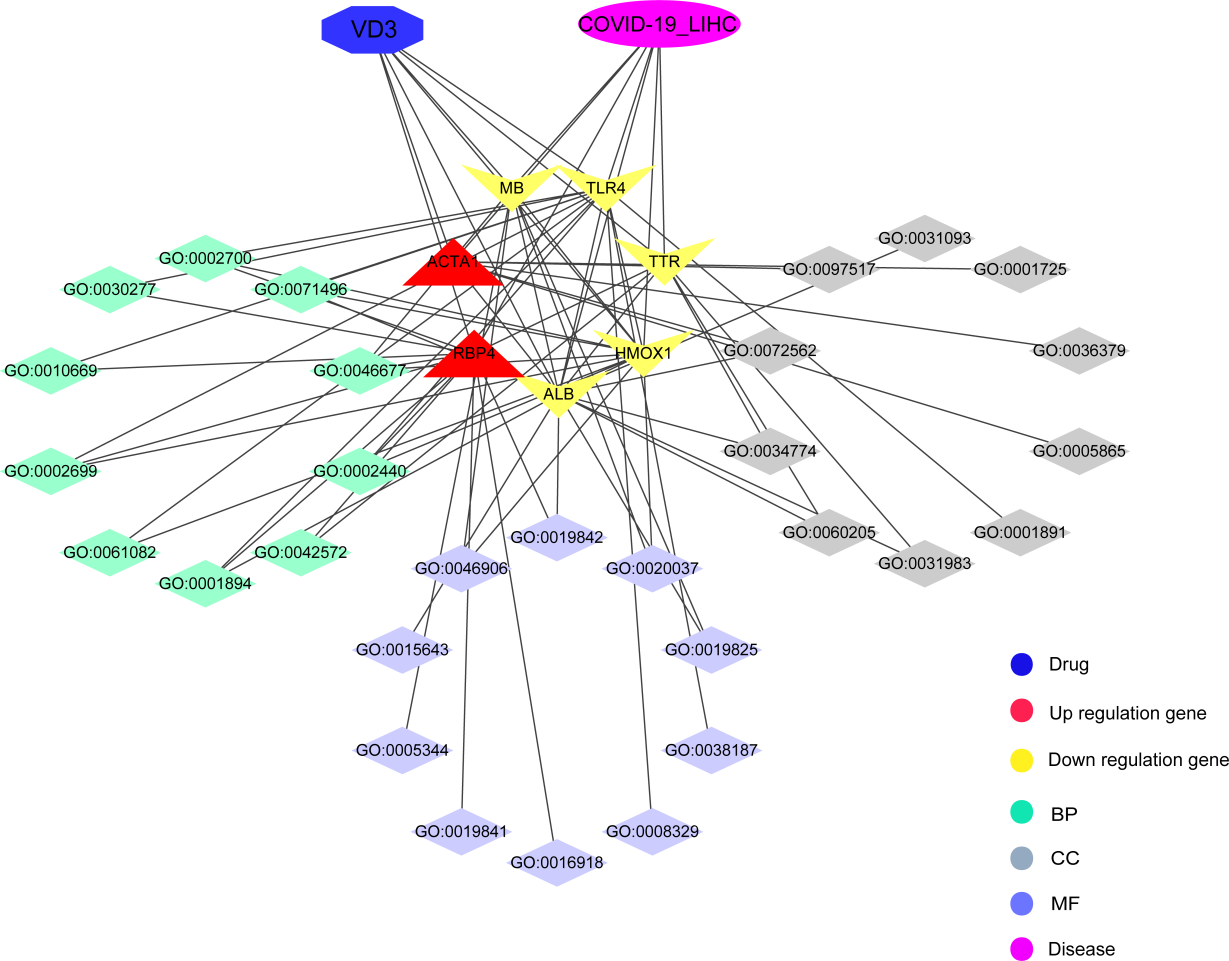
Interaction network for pharmacological targets and biological functions of vitamin D against COVID-19/HCC.

In summary, we identified many potential therapeutic targets of COVID-19/HCC and developed a reliable prognostic model for patients with HCC and COVID-19. Further, vitamin D may be used to treat COVID-19/HCC through the identified potential targets and pharmacological functions, including immunomodulation, anti-virus, anti-inflammation and so on (**Figure 10**). Moreover, the direct binding targets with high binding affinity of vitamin D against COVID-19/HCC were identified, which provided the evidence for the clinical application of vitamin D and rationale for subsequent clinical trials.

## Strengths and limitations

Notably, our study indicated the possible molecular mechanisms and potential pharmacological targets of vitamin D for treating COVID-19/HCC for the first time and provided some new insights into vitamin D in the treatment of COVID-19/HCC. Nevertheless, there remain a few limitations in our study need to be addressed. First, the findings of this study have not been validated in actual HCC patients with COVID-19; therefore, we need to collect real HCC patients with COVID-19 to verify these findings in the future. Second, the potential therapeutic use of vitamin D for COVID-19/HCC has not been validated experimentally, and further *in vivo* and *in vitro* experiments are needed to confirm the possible mechanisms and potential pharmacological targets.

## Data availability statement

The original contributions presented in the study are included in the article/**Supplementary Material**. Further inquiries can be directed to the corresponding author.

## Author contributions

YH and YY designed the study and wrote the manuscript. SC, DX, LX, XW, and WQ analyzed the results. BL helped to modified the manuscript. All authors contributed to the article and approved the submitted version.

## Funding

This work was supported by a grant from the National Natural Science Foundation of China (grant number 81902619).

## Conflict of interest

The authors declare that the research was conducted in the absence of any commercial or financial relationships that could be construed as a potential conflict of interest.

## Publisher’s note

All claims expressed in this article are solely those of the authors and do not necessarily represent those of their affiliated organizations, or those of the publisher, the editors and the reviewers. Any product that may be evaluated in this article, or claim that may be made by its manufacturer, is not guaranteed or endorsed by the publisher.
