# Supplementary material for: Identifying potential pharmacological targets and mechanisms of vitamin D for hepatocellular carcinoma and COVID-19
Source: Front Immunol. 2022 Aug 17;13:985781. doi: 10.3389/fimmu.2022.985781 (PMC9583923; doi:10.3389/fimmu.2022.985781)
Supplement: Supplementary file 1 [file DataSheet_1.pdf]

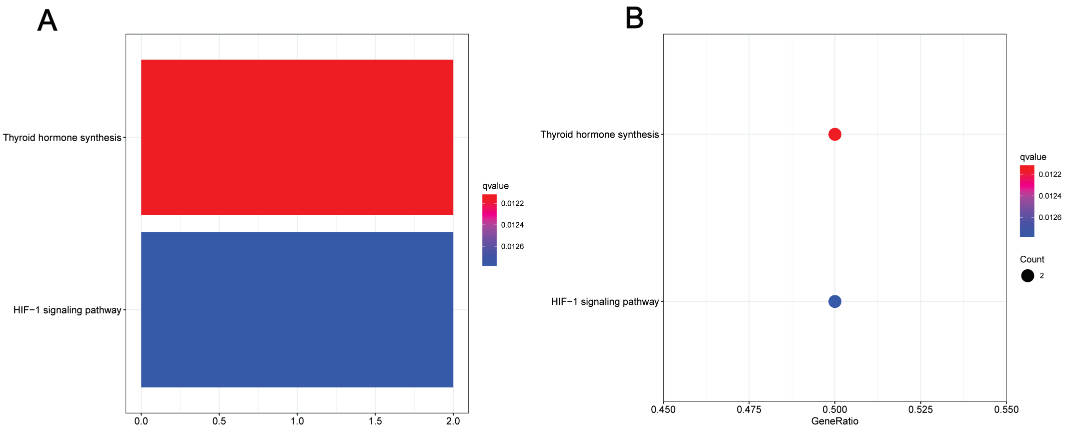

Figure S1. (A) Bar plot and **(B)** Bubble plot for KEGG enrichment analysis of vitamin D against COVID-19/HCC intersecting genes.
